# A mathematical model of brain glucose homeostasis

**DOI:** 10.1186/1742-4682-6-26

**Published:** 2009-11-27

**Authors:** Lu Gaohua, Hidenori Kimura

**Affiliations:** 1 Brain Science Institute, the Institute of Physical and Chemical Research (RIKEN) 2271-130 Anagahora, Shimoshidami, Moriyama-ku, Nagoya, 463-0003, Japan

## Abstract

**Background:**

The physiological fact that a stable level of brain glucose is more important than that of blood glucose suggests that the ultimate goal of the glucose-insulin-glucagon (GIG) regulatory system may be homeostasis of glucose concentration in the brain rather than in the circulation.

**Methods:**

In order to demonstrate the relationship between brain glucose homeostasis and blood hyperglycemia in diabetes, a brain-oriented mathematical model was developed by considering the brain as the controlled object while the remaining body as the actuator. After approximating the body compartmentally, the concentration dynamics of glucose, as well as those of insulin and glucagon, are described in each compartment. The brain-endocrine crosstalk, which regulates blood glucose level for brain glucose homeostasis together with the peripheral interactions among glucose, insulin and glucagon, is modeled as a proportional feedback control of brain glucose. Correlated to the brain, long-term effects of psychological stress and effects of blood-brain-barrier (BBB) adaptation to dysglycemia on the generation of hyperglycemia are also taken into account in the model.

**Results:**

It is shown that simulation profiles obtained from the model are qualitatively or partially quantitatively consistent with clinical data, concerning the GIG regulatory system responses to bolus glucose, stepwise and continuous glucose infusion. Simulations also revealed that both stress and BBB adaptation contribute to the generation of hyperglycemia.

**Conclusion:**

Simulations of the model of a healthy person under long-term severe stress demonstrated that feedback control of brain glucose concentration results in elevation of blood glucose level. In this paper, we try to suggest that hyperglycemia in diabetes may be a normal outcome of brain glucose homeostasis.

## Background

The concentration of blood glucose is controlled continuously through regulatory hormones, mainly insulin and glucagon. An increase in glucose concentration in the blood, for example after meals or under stress, increases insulin secretion and depresses glucagon secretion from the pancreas. The balanced counteraction of insulin and glucagon regulates glucose production from the liver and glucose conversion into fat and maintains blood glucose level within a relatively narrow range. Diabetes is generally considered as a peripheral disease characteristic of dysfunction of such peripheral glucose-insulin-glucagon (GIG) interactions.

In addition to peripheral GIG interactions, the recently recognized central brain-endocrine crosstalk also plays a critical role in glucose homeostasis [1]. The brain, on one hand, possesses its own glucose sensing machinery that protects itself from hypoglycemic injury by triggering a rapid secretion of counterregulatory hormones in response to low extracellular glucose levels [2]. On the other hand, repressive adaptation of glucose transport across the blood-brain-barrier (BBB) occurs in response to chronic hyperglycemia to prevent a rise in brain glucose content [3]. The physiological fact that maintenance of a constant brain glucose level is more important than that of blood glucose level suggests that the ultimate goal of the GIG regulatory system, which consists of peripheral GIG interactions and central brain-endocrine crosstalk, is homeostasis of glucose concentration in the brain rather than in the blood.

Correlated to the brain, psychological stress is also considered to have major effects on metabolic activity since energy mobilization is the primary result of fight-or-flight response. Stress stimulates the release of various hormones through the hypothalamus-pituitary-adrenal (HPA) axis and results in elevated blood glucose levels. Due to the same mechanism, stress may be a potential contributor to chronic hyperglycemia in diabetes. In particular, the disease and its medical treatments add further stress due to restriction on life style of diabetes. Although human studies on the role of stress in the onset and development of diabetes are few, a large body of animal research supports the notion that stress enhances the state of hyperglycemia in this disease [4].

Over the last 50 years, peripheral GIG interactions have been studied theoretically. Various mathematical models of glucose-insulin interaction have been developed after the first analogue model proposed by Bolie [5]. For example, Bergman and colleagues developed the so-called minimal model, which has been the model most applied in the current research on diabetes due to the small number of identifiable parameters used in the model [6]. Sturis et al. developed a mathematical model that uses three differential equations and one implicit time-delay to explore the physiological mechanism underlying ultradian oscillations in blood glucose and insulin concentrations [7]. Li et al. modified Sturis' model by introducing two explicit time-delays [8]. Excellent overviews of the mathematical models dealing with many aspects of diabetes are available, e.g., [9,10].

Although these models are useful either theoretically or practically, they lack key physiological aspects of the GIG regulatory system. That is, the roles of brain and stress are not included in any of these models. One of the major reasons is the general consideration that blood insulin is the main player in glucose homeostasis, while the brain and stress, which are participants in glucose homeostasis, are completely ignored in either physiology or clinical medicine. In fact, insulin is used as the sole drug in clinical practice to deal with blood hyperglycemia in diabetes. Another reason is related to the difficulty to represent stress quantitatively. To the best of our knowledge, quantitative measure of psychological stress has not yet been established.

The major purpose of this paper is to demonstrate the relationship between brain glucose homeostasis and peripheral blood hyperglycemia in diabetes. At the same time, this paper describes a theoretical model of the GIG regulatory system, in which the brain plays a major role, to provide a framework for quantitative discussion of the roles of brain and stress in glucose homeostasis both in normoglycemia and hyperglycemia.

For this purpose, the body is approximated compartmentally, while considering the brain as the controlled object and the body, with the exception of the brain, as the actuator. The concentration dynamics of glucose, as well as those of insulin and glucagon, are described in each compartment based on mass conservation law. The brain-endocrine crosstalk is modeled as a proportional feedback control that regulates hepatic glucose production and pancreatic hormonal secretion, together with the peripheral GIG interactions. Psychological stress, which is quantitatively represented by an abstract parameter in the model, introduces modification to the feedback control. A transfer function characteristic of gain and time constant is used to describe BBB adaptation to dysglycemia as a dynamic process.

The model is verified through comparison of its simulation profiles with the clinical data reported independently in various original studies. The model is subsequently used to simulate a healthy person under long-term severe stress with and without fast BBB adaptation. The relationship between brain glucose homeostasis and blood hyperglycemia are demonstrated by extensive simulations. Finally, theoretical discussion opens up the door for novel strategies for diabetes control.

## Hypothesis of brain glucose homeostasis

Glucose level in the brain mass is about 20% of that in the blood [11]. Control of brain glucose concentration is of supreme importance for human beings. Very low glucose concentrations can immediately induce seizures, loss of consciousness, and death, while chronic hyperglycemia would induce changes in hippocampal gene expression and function [12]. The range of brain glucose fluctuation is much smaller than that of blood glucose during euglycemia [13]. In rats, an increase of 50 mg/dl in blood glucose level from baseline value only caused 10 mg/dl increase in brain glucose level [14]. These physiological facts imply that the ultimate goal of the GIG regulatory system in the body, no matter whether it is healthy or not, may be not the homeostasis of glucose concentration in the blood, but the homeostasis of glucose concentration in the brain.

**Figure 1:**
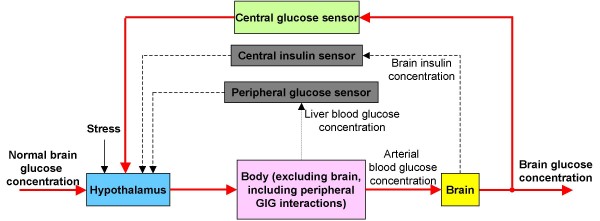
**Feedback control for brain glucose homeostasis**.

From the viewpoint of systems control, a one-to-one correspondence is established between the control of brain glucose homeostasis and a servo-mechanical control system, as shown in Fig. 1. In this framework of glucose homeostasis, the brain is the controlled object and the brain glucose concentration is the controlled variable, while the rest of the body is considered as the actuator, where peripheral GIG interactions function under the influence of brain-endocrine crosstalk.

Such a framework corresponding to feedback control of brain glucose homeostasis is supported by anatomical evidences. Various glucosensing neurons are located in an interconnected network distributed throughout the brain, which also receives afferent neural input from glucosensors in the liver, carotid body, and small intestines [15]. Central insulin is also a hormonal signal that provides negative feedback to the brain for the regulation of glucose homeostasis [16]. After receiving information (central and peripheral glucose, central insulin) from afferent nerves, the hypothalamus sends signals, by stimulating the autonomic nerves or by releasing hormones from the pituitary gland, to the peripheral organs, including the liver and pancreas, to maintain homeostasis [17].

As shown in Fig. 1, stress can be viewed as a disturbance input to the controller. Therefore, various efferent signals from the controller to the actuator are affected by stress before regulating hepatic glucose production and pancreatic hormonal secretion.

## Methods

### Model development

#### Assumptions

In order to establish a mathematical model of brain glucose homeostasis, some assumptions are unavoidable. The main assumptions include,

(1) The human body is composed of various segments, each of which consists of homogeneous mass and/or blood compartments;

(2) All parameters are time-invariant, such as constant blood flow into the blood compartment of each segment, constant distribution volumes for glucose, insulin and glucagon in each compartment;

(3) Hepatic glucose production and pancreatic hormonal secretion are regulatory methods of blood glucose. There are no limitations in these processes;

(4) Glucose is utilized in tissue mass compartment or red blood cells, while insulin and glucagon are cleared in tissue mass compartments only;

(5) Both hepatic glucose production and pancreatic hormonal secretion depend on local state of the liver mass and pancreas mass. The same is true for glucose utilization or hormone removal in the tissue mass compartment.

(6) The GIG regulatory system is independent of other physiological functions.

Other less important assumptions are made in the text when necessary.

#### Model structure

In an integrative model developed by the authors previously for systems medicine in the intensive care unit, the body is approximated by 6 segments (cranial, cardiocirculatory, lungs, muscle, visceral and others) or 13 compartments, and various parameters are determined mainly from the literature [18-21].

The compartmental structure and its parameters are applied in this study. Since the visceral segment in the original model represented a set of visceral tissues, including the liver, kidneys, gut, and pancreas, it is necessary to describe this visceral segment in detail in order to take account of hepatic glucose production, pancreatic hormonal secretion, gastrointestinal glucose absorption and glucose loss via urine in case of hyperglycemia.

**Figure 2:**
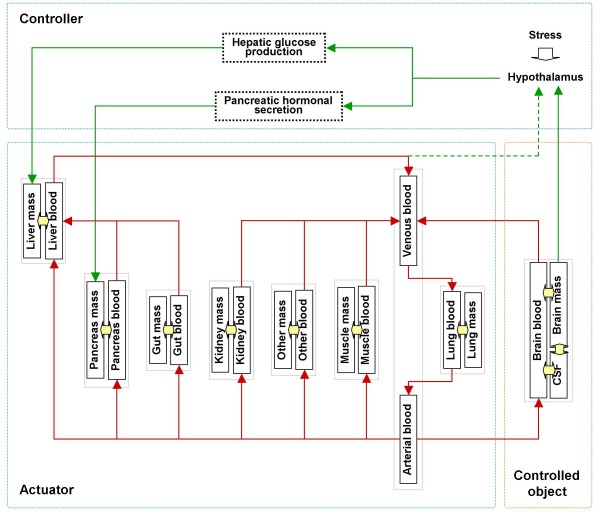
**Compartment model of brain glucose homeostasis**.

As shown in Fig. 2, the extended model consists of 9 segments or 19 compartments. The cranial segment consists of 3 compartments, corresponding to the brain mass, blood and cerebrospinal fluid (CSF). The cardiocirculatory segment is composed of arterial and venous compartments. Each of the other 7 extracranial segments comprises 2 compartments, that is, one is the mass and the other is the blood.

Based on the anatomy of the hepatic portal vein, blood flow from the pancreas segment and that from the gut segment enter the blood compartment of the liver segment and then join the systemic circulation, together with the hepatic arterial blood flow from the arterial part of the cardiocirculatory segment.

Glucose, insulin and glucagon in the blood circulate through various blood compartments, which transact with their adjacent mass compartments. Glucose is produced endogenously in the liver mass compartment, or given exogenously into the gut mass or venous blood compartment. Insulin is produced in the pancreas mass or infused into the muscle mass or venous blood compartment. By contrast, glucagon is generated only in the pancreas mass compartment.

Glucose is utilized in all tissue mass compartments and in the arterial compartment by erythrocytes, while insulin and glucagon are cleared in tissue mass compartments only.

One of the main differences between the current extended model and the original model is that the former considers the cranial segment as the controlled object, the rest of the body as the actuator, and arterial blood glucose concentration as the actuating signal.

Another difference is the introduction of a feedback control loop of brain glucose in the extended model. Neuronal and hormonal signals are generated based on the brain glucose state and modified by stress before they regulate hepatic glucose production and pancreatic hormonal secretion. Such feedback loop of brain-endocrine crosstalk contributes to the control of brain glucose homeostasis, together with the peripheral GIG interactions occurring in the actuator.

Mathematical descriptions of the controlled object, the actuator and the controller are given separately during modeling.

### Model of controlled object

#### Governing equations

Applying the mass conservation law, the dynamics of glucose in the cranial segment is described mathematically as follows,

where **V **denotes the diagonal matrix (3 × 3) of distribution volume, **G **vector (3 × 1) of glucose concentration, *G*_*art *_is the glucose concentration in the arterial blood flowing into the brain blood compartment of the cranial segment. The left hand side represents the storage rate of glucose. Various matrices and vectors are given as follows,

The superscript *T *denotes transposition of the vector. All symbols, _subscripts _and ^superscripts ^are summarized in the Glossary. From the viewpoint of systems control,  is considered as the controlled variable (output) and *G*_*art *_as the controlling input.

#### Detailed description of the nonlinear term F in Equation (1)

##### Metabolism

Brain cells use glucose without the intermediation of insulin [22]. Such insulin-independent glucose utilization in the brain mass, *M*^*brain*^, is assumed a function of brain glucose concentration, as described by the following Michaelis-Menten equation,

where *m*_1 _and *m*_2 _are estimable parameters. However, neither *m*_1 _nor *m*_2 _is currently available from reported data by the authors, as it is based on glucose concentration in the brain mass, but not in the blood. For simulation purpose, it is assumed in this model that  and , on the basis of brain glucose concentration at the steady-state . Then equation (2) is modified to,

##### Facilitated transport through BBB/BCB

**Figure 3:**
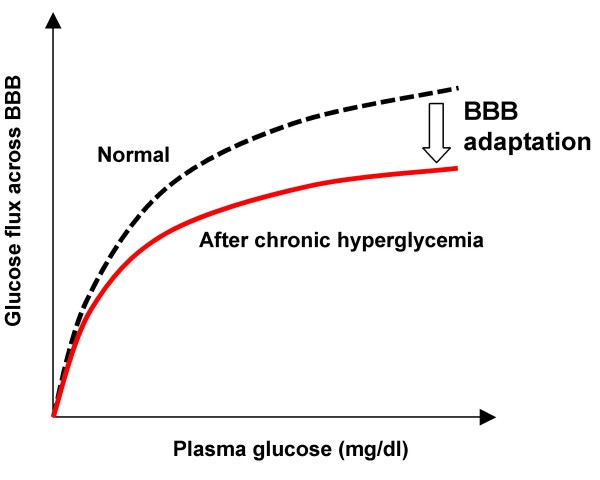
**Facilitated glucose transport across BBB**.

The blood-brain-barrier (BBB) and blood-CSF barrier (BCB) are the interfaces between the brain blood and brain mass. Physiologically, the BBB and BCB help to maintain brain glucose homeostasis by regulating the facilitated saturable transport of glucose with their semi-impermeability, as shown in Fig. 3.

According to Rapoport [23], glucose transport across the BBB, , is described by the Michaelis-Menten equation with two parameters, the maximal transport rate (*T*_*G*0_) and Michaelis constant (*F*_*G*0_), as follows,

In this model, *K*_*G*0 _= 0.9 [*mg*/*ml*] (Rapoport 1976) and then .

The facilitated infusion of glucose across the BCB, , is described similarly, although it is minor.

#### Dynamic BBB/BCB adaptation

Various clinical observations suggest that the dynamics of glucose transport across BBB/BCB is influenced by the adaptive nature of the barriers. For example, experimentally-induced chronic hypoglycemia in rats elicited overexpression of glucose transporter-1 (GLUT-1) and redistribution of GLUT-1 at the BBB [24]. Overexpression of GLUT-1 is viewed to have a positive effect on the maximal transport rate, *T*_*G*0_, without altering the Michaelis constant, *K*_*G*0 _[25]. In rats with chronic hyperglycemia, the maximum glucose transport capacity of the BBB decreased from 400 to 290 micromoles per 100 grams per minute, and the glucose transport rate in the brain decreased to 20 percent below normal when plasma glucose was lowered to normal values [3].

This mechanism, termed BBB adaptation to chronic hyperglycemia in Fig. 3, represents a dynamic process with a long time constant, since brain glucose transport is not altered following short episodes of recurrent hypoglycemia in healthy human volunteers [25]. The adaptation must be inactive within the euglycemic range, since frequent variations, known as ultradian oscillations, occur in blood glucose.

Therefore, a first-order dynamics of two parameters, namely, gain and time constant, is introduced into this model to modify the maximal glucose transport rate *T*_*G*0 _with respect to blood dysglycemia as follows,

where Δ*T*_*G *_is the response of maximal glucose transport rate *T*_*G *_with respect to hyperglycemia (,  is the maximum value of glucose concentration in the brain blood compartment at the steady state) or hypoglycemia (,  is the minimum value of glucose concentration in the brain blood compartment at the steady state). *κ*_*G *_and *τ*_*G *_denote gain and time constant, respectively. *s *in equation (5) is LapLace operator.

Equation (3) describing the facilitated infusion through BBB/BCB is thus modified to,

where *T*_*G *_is adaptable according to equations (4) and (5) with respect to dysglycemia in the brain blood.

### Model of the actuator characteristic of peripheral GIG interactions

#### Governing equations

The dynamics of glucose concentration in each extracranial segment (with the exception of the cardiocirculatory segment) is described as follows,

where *V *denotes glucose distribution volume and *G *glucose concentration. The superscript *x *represents the segment while the subscript *mass *or *blood *represents the mass or blood compartment, respectively.  denotes glucose diffusion from the blood compartment into its adjacent mass compartment in segment *x*.  and  denote glucose production and utilization in the mass compartment of segment *x*. *w*^*x *^is blood flow. *G*_*art *_is glucose concentration in the arterial blood. The last term on the right hand side of equation (7) represents net glucose delivery by blood flow into the blood compartment of segment *x*.

The term *p *in equation (6) only appears in the mass compartment of the liver segment due to the endogenous hepatic glucose production or in the mass compartment of the gut segment due to the exogenous gastrointestinal glucose absorption. The term *u *in equation (6) consists of the two parts, namely, insulin-independent utilization and insulin-dependent utilization. The former corresponds to glucose discard from the kidney mass compartment through the urine in hyperglycemia, while the latter is mostly due to glucose metabolism in the muscular mass and various visceral mass compartments.

In the arterial and venous compartments of the cardiocirculatory segment, the dynamics of glucose concentrations are given as follows,

where *V*_*art *_and *V*_*ven *_denote the distribution volumes of glucose in the arterial and venous compartments of the cardiocirculatory segment. *G*_*art *_and *G*_*ven *_are the glucose concentrations in these two compartments. *w *is total cardiac output, while *W*^*y *^represents blood flow from the blood compartment of segment *y *(including the cranial, liver, kidneys, muscle and the other segment), particularly, .  is the blood glucose concentration in segment *y*, in particular,  denotes glucose concentration in the blood compartment of the lung segment. The term  in equation (8) describes the insulin-independent glucose utilization by erythrocytes, which is assumed to occur only in the arterial compartment for simplification, although the glucose is physiologically utilized by erythrocytes in all blood compartments. The term of  in equation (9) denotes exogenous glucose infusion into the venous blood.

### Explanation of various terms in equations (6)-(9)

#### Hepatic glucose production

Conversion of glucose into glycogen, as well as glycogenolysis and/or gluconeogenesis, in the liver is one of the primary strategies involved in the regulation of blood glucose concentration. High levels of either glucose or insulin serve to reduce glucose production by the liver, while glucagon stimulates hepatic glucose production.

The action of insulin on glucose production is a reflection of insulin concentration in the extracellular space, rather than in blood [26]. Therefore, hepatic glucose production depends not on the concentrations of glucose, insulin and glucagon in the blood compartment, but rather on their concentrations in the mass compartment of the liver segment.

The following linear equation is introduced to describe hepatic glucose production phenomenologically,

where  denotes net hepatic glucose production and  is its steady-state value. Positive  means net glucose is produced by the liver while negative  means net glucose is stored or degraded in the liver. ,  and  are local concentrations of glucose, insulin and glucagon in the hepatic mass compartment, respectively. ,  and  are their respective steady-state values. *k*_1_, *k*_2 _and *k*_3 _are positive parameters to be estimated. The four terms in the brackets correspond to basal production, contribution of hepatic glucose state, that of hepatic insulin state and that of hepatic glucagon state, respectively, to hepatic glucose production based on their steady-states. The signs of addition and subtraction are based on physiological functions concerning the effects of glucose, insulin and glucagon on hepatic glucose production.

Equation (10), which is based on the concentrations of glucose, insulin and glucagon in the hepatic mass compartment, is completely different from mathematical descriptions based on their concentrations in the blood, which are commonly used in the currently existing theoretical models. Therefore, it is difficult to determine the numerical values for parameters *k*_1_, *k*_2 _and *k*_3 _from the literature. The values of these parameters are chosen based on trial-and-error during model verification and improvement.

#### Utilization by peripheral tissue

The action of insulin necessary to stimulate peripheral glucose utilization is also determined by its concentration in interstitial fluid that bathes insulin-sensitive cells [27]. The uptake of glucose peripherally (primarily the muscle, gut, lungs, liver, pancreas, kidneys and the other mass) depends not only on local glucose concentration but also on local insulin concentration. A suitable form of this utilization in the peripheral segment *x *is given as follows,

where  denotes glucose utilization by the mass compartment in segment *x*,  is its steady-state value. *k*_4 _and *k*_5 _are estimable positive parameters. The two brackets on the right hand describe the contributions of local glucose state and local insulin state to glucose utilization. Since no data are available to the authors from the literature, both the value of parameter *k*_4 _and that of *k*_5 _are estimated by trial-and-error.

#### Utilization by erythrocytes

Similar to brain glucose metabolism, glucose utilization by erythrocytes, *u*^*red *^in equation (8), is independent of insulin (and glucagon) concentration, but dependent on glucose concentration. It is a function of arterial blood glucose concentration with saturation. Following this perspective, the form of this contribution in equation (8) is described by the following equation (12).

where  is glucose utilization by erythrocytes,  is its steady-state value. *G*_*art *_denotes glucose concentration in the arterial blood, and *G*_*art*0 _is its steady-state value.

#### Loss through urine

Glucose uptake by the kidneys consists of the following two types. First, insulin-glucose-dependent metabolism occurs in the kidney mass compartment, as given by equation (11). Second, increased blood glucose concentration leads to loss of glucose through urine. Such glucose loss is independent of insulin and glucagon. On account of the physiological fact that glucose appears in the urine when the blood glucose concentration is over 1.8 mg/ml [22], it is assumed that, when glucose concentration in the kidney mass compartment is below a threshold level (for simplification, 1.8 mg/ml in the model), urinary glucose loss is zero. In contrast, the rate of urinary glucose loss increases linearly with increasing concentration in the kidney mass compartment, once the glucose concentration in the kidney mass compartment exceeds the threshold level. Mathematically, this is described by the equation,

where  is the urinary glucose loss, *w*^*urine *^is urinary flow, at an average of 2 liters per day (0.023 ml/s),  is the glucose concentration in the mass compartment of the kidney segment.

#### Permeability via capillary bed

The transcapillary delivery of glucose between the blood compartment and its adjacent mass compartment depends on the permeability coefficient and the concentration difference between the two compartments.

Assuming that the relation between transcapillary delivery of glucose and the concentration difference is linear, the term  in equations (6) and (7) is described by,

where  is the permeability coefficient of glucose between the mass and blood compartments.

At steady-state, metabolic utilization of glucose in each mass compartment of the extracranial segments should be equal to the net glucose transport from its adjacent blood compartment. Accordingly, it is possible to estimate the permeability coefficient  from the metabolic glucose utilization (), steady-state glucose concentrations in the mass compartment and the blood compartment, as given by the following equation (15).

However, the transcapillary delivery of glucose is under the influence of blood insulin [28]. Therefore, the glucose diffusion  in equation (14) is modified as follows,

where the contribution of local insulin state to transcapillary glucose delivery is taken into account by introducing the last brackets.

### Model of insulin and glucagon dynamics

#### Insulin dynamics

The concentration dynamics of insulin in segment *x*, representatively consisting of mass and blood compartments, is described by dynamic mass balance as follows,

where  and  are distribution volumes of insulin,  and  are insulin concentration in the mass and blood compartments of segment *x*, respectively,  is insulin transport from the blood compartment to its adjacent mass compartment in segment *x*, *I*_*art *_denotes the arterial insulin concentration,  is the production rate of insulin,  is insulin removal from the mass compartment in segment *x*, and *w*^*x *^is blood flow to segment *x*. The last term on the right hand side of equation (17) represents net insulin delivery through blood flow into the blood compartment of segment *x*.

Endogenous insulin is secreted from beta-cells in the pancreatic mass. Both elevated blood glucose and glucagon stimulate insulin secretion [22]. It is reasonable to consider that the concentrations of glucose and glucagon in the pancreatic mass determine the level of endogenous insulin production. The following equation mathematically describes pancreatic insulin secretion,

where  is insulin secretion within the pancreatic mass compartment,  is its steady-state value, and *k*_6 _and *k*_7 _are estimable positive parameters. Negative calculated  is set to zero because of its physiological meaningless. The three terms in the brackets correspond to basal secretion, contribution of pancreatic glucose state and that of pancreatic glucagon state, respectively, to pancreatic insulin secretion based on their steady-states. The plus signs are based on the physiological functions concerning effects of glucose and glucagon on pancreatic insulin secretion. The values for parameters *k*_6 _and *k*_7 _are unavailable in the literature and given by the authors based on trial-and-error.

Since insulin is cleared by all insulin-sensitive tissues,  is dependent on the local concentration of insulin in each of the extracranial mass compartments. Therefore,

where  denotes rate of insulin removal from the mass compartment of segment *x*,  is its steady-state value, and *k*_8 _is estimable positive parameter. The bracket on the right hand describes the contribution of local insulin state to insulin removal. Value of parameter *k*_8 _is also not available in the literature.

As no insulin is produced in the brain, intracranial insulin concentrations depend on BBB/BCB transport of peripheral insulin. However, such transport is characterized by saturation [16]. Furthermore, hyperglycemia abolishes insulin transport across BBB [16]. Therefore, it is reasonable in this model to consider that BBB/BCB insulin transport also adapts with respect to dysglycemia.

Like that of glucose, insulin transport from brain blood to brain mass or to CSF also follows the Michaelis-Menten equation mathematically, on account of the analogous blood-brain barrier transport systems existing for glucose, amino acids, plasma proteins, as well as the circulating insulin [29]. Altogether, a formula similar to equation (3) is introduced to describe the facilitated transport across BBB/BCB of insulin as follows,

where *K*_*I*0 _is the Michaelis constant for the facilitated insulin diffusion across BBB/BCB, and *T*_*I *_is the maximal transport rate of insulin across BBB/BCB, which is glucose dependent, as given by equations (4) and (5), as follows,

where *T*_*I*0 _is the steady-state value of *T*_*I*_. Δ*T*_*I *_is the response of maximal insulin transport rate *T*_*I *_with respect to hyperglycemia (,  is the maximum value of glucose concentration in the brain blood compartment at the steady state) or hypoglycemia (,  is the minimum value of glucose concentration in the brain blood compartment at the steady state). Similar to glucose transport into brain mass and CSF compartments, *κ*_*I *_and *τ*_*I *_are gain and time constant, respectively. The time constant *τ*_*I *_should be some days in the rat and some years in human. Both gain *κ*_*I *_and time constant *τ*_*I *_are individual dependent.

In the extracranial segments, the transcapillary delivery of insulin from the blood compartment to its adjacent mass compartment is mediated through passive diffusion [28].

Thus,

where  is permeability coefficient of insulin between the mass and blood compartments.

Since the metabolic removal of insulin in all insulin-sensitive mass compartments of the extracranial segments is equal to insulin diffusion from their adjacent blood compartments,  could be determined from metabolic insulin removal, insulin concentrations in the mass compartment and blood compartment at steady-state, as follows,

The dynamics of insulin concentrations in the cranial segment are represented mathematically similar to equation (1), while the dynamics of insulin concentrations in the arterial and venous compartments of cardiocirculatory segment are described by using mathematical equations similar to equations (8) and (9).

#### Glucagon dynamics

Similar to that of glucose and insulin, the concentration dynamics of glucagon in each segment *x *consisting of the mass and blood compartments is described as follows,

where  and  are the distribution volumes of glucagon,  and  are the glucagon concentrations of the mass and blood compartments in segment *x*, respectively,  is glucagon transport from the blood compartment to its adjacent mass compartment in segment *x*, *E*_*art *_is the arterial glucagon concentration,  and  denote glucagon production and removal from the mass compartment in segment *x*, respectively, and *w*^*x *^is the blood flow to segment *x*. The last term on the right hand side of equation (26) represents net glucagon delivery through blood flow into the blood compartment within segment *x*.

In case of glucagon dynamics, the term  corresponds to glucagon production from alpha-cells in the pancreatic mass. It depends on the concentrations of glucose and insulin in the pancreatic mass. In other words, either elevated level of glucose or that of insulin depresses pancreatic glucagon secretion. Its mathematical description is given by:

where  is glucagon secretion in the pancreatic mass compartment,  is its steady-state value, and *k*_9 _and *k*_10 _are estimable positive parameters. Similar to that of , negative calculated  is set to zero. Three terms in the brackets correspond to the basal secretion, contribution of pancreatic glucose state and that of pancreatic insulin state, respectively, to pancreatic glucagon secretion based on their steady-states. The minus signs are based on the physiological functions describing the effects of glucose and insulin on pancreatic glucagon secretion. Values of parameter *k*_9 _and *k*_10 _are not available in literature.

Glucagon is degraded in all extracranial mass compartments, mainly by the kidney and the liver. Compared to extracranial glucose metabolism and insulin removal,  is assumed to be independent of ambient glucose or insulin concentrations. That is, the term  is a function of local glucagon concentration only, as given by,

where *v*_*E *_denotes degradation constant, which is determined from the steady-state values of glucagon concentration () and glucagon removal () in the mass compartment,

The dynamics of glucagon concentrations in the arterial and venous compartments of the cardiocirculatory segment are described by using mathematical descriptions similar to equations (8) and (9).

### Brain-endocrine crosstalk

As mentioned above, the GIG regulatory system comprises the peripheral GIG interactions and the central brain-endocrine crosstalk. The former is described mathematically in detail during modeling the actuator in equations (10), (11), (18) and (19). The latter, mainly consisting of the glucosensor-hypothalamus-liver-pancreas link [17,30], modifies the peripheral GIG interactions in order to control brain glucose homeostasis, as shown in Fig. 1.

Various glucosensing neurons are distributed throughout the brain, which also receives afferent neural inputs from glucosensors in the liver, carotid body, and small intestines [15]. It is considered that glucose concentration in the brain mass is the major signal to the hypothalamus for the regulation of brain glucose homeostasis, while central insulin is a hormonal signal that provides negative feedback to the brain. For example, an increase in insulin signal in the hypothalamus elicits responses that reduce hepatic glucose production [31]. The anatomy of the brain-endocrine crosstalk is described in detail by Uyama and colleagues [17].

However, the current knowledge regarding neuronal and hormonal signals for hepatic glucose production and pancreatic hormonal production are not quantitative but rather qualitative in nature. For simplification of theoretical discussion, it is assumed that a proportional feedback control of brain glucose occurs, mainly based on differences between brain glucose concentration and baseline. As shown in Fig. 1, peripheral glucose and central insulin act as auxiliary input to the central hypothalamic controller.

The controlled error is given as follows,

where *a*_*cg*_, *a*_*ci *_and *a*_*pg *_are parameters with appropriate unit. They are adjustable in the model to reflect varying importance of various glucose and insulin sensors in monitoring the glucose state in the body. The three brackets on the right hand describe the contributions of central glucose state, central insulin state and peripheral glucose state. The two plus signs (+) in equation (30) are based on the physiological fact that glucose-sensing neurons in the brain serve as integrators of various metabolic signals [13].

Based on the assumption of proportional feedback control of brain glucose, three efferent signals are generated by the hypothalamus, that is,

where *α*, *β *and *γ *denote signals regulating hepatic glucose production, pancreatic secretion of insulin and glucagon, respectively, *k*_*γ*_, *k*_*β *_and *k*_*α *_are parameters with appropriate units. They are all adjustable in the model to reflect varying importance of various mechanisms involved in glucose homeostasis. Since insulin depresses both hepatic glucose production and pancreatic glucagon secretion, *k*_*γ *_> 0 and *k*_*a *_> 0, while *k*_*β *_> 0.

Each of these signals acts to modify hepatic glucose production, pancreatic secretion of insulin and glucagon. Therefore, equations (10), (18) and (27) are changed to,

Where ,  and  denote hepatic glucose production, pancreatic insulin secretion and pancreatic glucagon secretion, respectively. ,  and  are steady-state values. The first bracket on the right hand of each equation describes the effect of brain-endocrine crosstalk.

### Stress input to the central controller

To take into consideration the effect of psychological stress, it is necessary to quantify it. To the best of our knowledge, a quantitative measure of stress has not been established. Therefore, a rather abstract variable of positive value varying between 0 and 1 is introduced to describe mild to severe stress, respectively. Various durations of stress, namely, short-term, repeated, long-term, are also used to describe the stress encountered in daily life.

It is well documented that stress causes a direct increase in pancreatic glucagon production through catecholamines, which play a critical role in these fight-or-flight circumstances [32]. The increase in glucagon levels, stimulated by increased catecholamine, drives increased glycogenolysis and gluconeogenesis in liver. In contrast, insulin is decreased during times of stress [32,33]. The fight-or-flight response to stress characteristically increases hepatic glucose production.

Taking account of the systemic effects of stress on the hepatic glucose production and pancreatic hormonal secretion, various coefficients can be introduced into equations (10'), (18') and (27') to describe the total effects of stress. That is,

where *s *denotes the severity of stress. The first bracket on the right hand of each equation describes the effect of stress.

### Parameters

Various physiological parameters, such as distribution volume, blood flow and metabolic allocation, are common to the current model and the integrated model developed previously for systems medicine in the intensive care [18-21]. In addition to the original data applicable in this model, values of special parameters for the GIG regulatory system are added mainly based on literature.

#### Production or secretion

Glucose input into the circulation is normally approximately 2 mg/min/kg of body weight [34]. At normal fasting level of blood glucose, the rate of insulin secretion is in the order of 25 ng/min/kg of body weight [22]. The secretion rate of glucagon could be estimated based on glucagon removal under steady-state conditions. Such estimation yields a value of approximately 1400 pg/min/kg of body weight in humans [35].

#### Utilization or removal

The main contributors to glucose disappearance in the fasting, resting state are brain, lean tissues, adipose tissue and red blood cells [34]. In the model, total glucose input of 2.24 mg/s is assumed to be utilized in various mass compartments and the arterial blood compartment (brain 0.65, lung 0.08, pancreas 0.01, gut 0.25, liver 0.12, kidney 0.02, muscle 0.67, the residual 0.22, and the arterial compartment 0.22 mg/s). The allocation of glucose utilization is completely assumed while taking account of the weight of various mass compartments.

Under normal physiological state, insulin secreted by the pancreas is cleared in the liver, kidney, muscle, adipose tissue and other tissues [32]. As in case of glucose utilization, total insulin secretion of 28 ng/s is considered to be cleared in various tissues (brain 0.27, lung 3.70, gut 0.26, liver 21.43, kidney 0.79, muscle 0.74, and the residual 0.81 ng/s).

Both the kidney and liver also remove glucagon from the circulation, accounting for 30% and 20% of disposal, respectively [32]. The total glucagon secretion of 1569.73 pg/s is cleared in various mass compartments (brain 19.42, lung 12.23, gut 39.80, liver 470.62, kidney 313.75, muscle 390.91, and residual 323.0 pg/s).

#### Concentration

In normal subjects, glucose concentration in the brain is about 20 mg/dl, CSF 60 mg/dl, jugular venous blood 90 mg/dl and carotid arterial blood 100 mg/dl [11]. In the present model, they are assumed to be 18.42, 58.42, 88.42, and 96.75 mg/dl, respectively. The glucose concentrations in various mass compartments are assumed on a mass-to-blood glucose level of about 60% [36]. Particularly, it is possible to calculate the concentration in various blood compartments according the steady-state values of glucose concentration and glucose utilization in a mass compartment without glucose production as follows,

Insulin concentration in the brain is less than that in the blood [37]. Insulin concentrations in CSF, brain mass and arterial blood are assumed to be 2.83, 2.41 and 43.56 ng/dl, respectively, in the model, while considering that the insulin permeation across BBB/BCB into the CSF and brain mass is only removed from the brain mass. Insulin concentrations in various extracranial mass compartments are assumed on account of mass-to-blood insulin level of about 25% [27].

In the model, the arterial blood glucagon concentration is 0.81 pg/dl, which is consistent with the physiological range of blood glucagon concentration of between 0.25 and 1.5 pg/dl after 12 h fast [35]. Glucagon concentrations in various mass and blood compartments are assumed on account of mass-to-blood glucose level of about 40%, taking account of the physiological fact that the plasma protein concentration in the interstitial space is normally about 40% of that in the plasma [38].

Furthermore, the concentrations of insulin and glucagon in various blood compartments could be calculated as in case of glucose.

#### Permeability coefficient

The permeability coefficient at the capillary bed could be estimated by equations (15), (24) and (29), based on the steady-state value of local concentrations and metabolic utilization or removal. The estimated values are confirmed by a few available clinical parameters. For example, the permeability coefficient in muscle is 1.7-6.0 ml/min/100 g for glucose and 0.5 ml/min/100 g for insulin [28].

As assumed above, all these parameters are time-invariant. Various physical and physiological parameters used for simulations are summarized in Table 1, Table 2 and Table 3.

**Table 1:** Parameters characterizing the GIG regulatory system: Concentration

Segment	Compartment	Volume (ml)*	**Glucose concentration (mg/ml)**^&^	**Insulin concentration (ng/ml)**^&^	**Glucagon concentration (pg/ml)**^&^
Brain	CSF	150	0.584	0.028	8.02
	mass	1374	0.184	0.024	8
	blood	6.0	0.884	0.402	78.614
Lung	mass	1669	0.591	0.080	16.001
	blood	70	0.971	0.436	81.120
Pancreas	mass	70	0.662	150.672	27985.9
	blood	0.29	0.962	27.115	1575.13
Gastrointestinal	mass	3041	0.639	0.087	15.995
	blood	12.4	0.939	0.407	76.705
Liver	mass	1541	1.304	0.182	18.027
	blood	6.28	1.104	0.902	159.225
Kidney	mass	253	0.668	0.052	11.554
	blood	1.03	0.968	0.372	55.701
Muscle	mass	22023	0.611	0.061	16.767
	blood	101	0.951	0.377	57.982
Residual	mass	27668	0.847	0.081	20.747
	blood	125	0.914	0.377	49.921
Cardiocirculatory	arterial	1129	0.968	0.436	81.104
	venous	3609	0.973	0.497	81.324

*: [21]

&: [32]. Estimated on base of compartmental mass.

**Table 2:** Parameters characterizing the GIG regulatory system. Consumption

Segment	Compartment	Flow (ml/s)*	**Glucose consumption (mg/s)**^&^	**Insulin consumption (ng/s)**^&^	**Glucagon consumption (pg/s)**^&^
Brain	CSF	0	--	--	--
	mass	0	0.65	0.27	19.42
	blood	13	--	--	--
Lung	mass	0	0.08	3.70	12.23
	blood	100	--	--	--
Pancreas	mass	0	0.01	0.0	0.0
	blood	1.75	--	--	--
Gastrointestinal	mass	0	0.25	0.26	39.80
	blood	15.75	--	--	--
Liver	mass	0	0.12	21.43	470.62
	blood	22.523	--	--	--
Kidney	mass	0	0.02	0.79	313.75
	blood	20.5	--	--	--
Muscle	mass	0	0.67	0.74	390.91
	blood	21	--	--	--
Residual	mass	0	0.22	0.81	323.0
	blood	23	--	--	--
Cardiocirculatory	arterial	100	0.22	--	--
	venous	100	--	--	--

*: [21]

&: [32]. Estimated on base of compartmental mass.

**Table 3:** Parameters characterizing the GIG regulatory system: Permeability

Segment	Compartment	**Glucose permeability (ml/s)**^&^	**Insulin permeability (ml/s)**^&^	**Glucagon permeability (ml/s)**^&^
Brain	CSF	0.022 (csf-mass)	1.734 (csf-mass)	9.711 (csf-mass)
	mass	(*K *= 0.9 mg/ml)	(*K *= 10.59 ng/ml)	(*K *= 2118 pg/ml)
	blood	--	--	--
Lung	mass	0.061	0.341	0.191
	blood	--	--	--
Pancreas	mass	0.006	0.227	0.063
	blood	--	--	--
Gastrointestinal	mass	0.253	1.111	0.622
	blood	--	--	--
Liver	mass	11.013	19.454	3.735
	blood	--	--	--
Kidney	mass	0.021	21.885	7.353
	blood	--	--	--
Muscle	mass	2.501	11.852	6.637
	blood	--	--	--
Residual	mass	0.747	9.016	5.049
	blood	--	--	--
Cardiocirculatory	arterial	--	--	--
	venous	--	--	--

&: Estimated on base of equations (15), (24) and (29).

#### Suggested values for some unavailable parameters

Values of some parameters are assumed during model verification and model improvement, since no data are available in the literature, as mentioned above. Calculations of these parameters are also based on the assumption that hepatic glucose production, pancreatic hormonal secretion, glucose utilization and hormonal removal depend on local concentrations of glucose, insulin and glucagon in the mass compartments, as described in equations (10), (11), (12), (13), (18), (19), (27) and (28), in the present model. Such assumption is acceptable in a mathematical approach because the shapes of the functions, instead of their mathematical forms, are more important [8]. Particularly, the following values of appropriate units are assumed (Table 4) in the model for simulation.

**Table 4:** Other assumed parameters

Symbol	Description	Assumed value
*k*_1_	Positive parameter concerning contribution of hepatic glucose state to hepatic glucose production in Equation 10	1.0
*k*_2_	Positive parameter concerning contribution of hepatic insulin state to hepatic glucose production in Equation 10	0.005
*k*_3_	Positive parameter concerning contribution of hepatic glucagon state to hepatic glucose production in Equation 10	0.25
*k*_4_	Positive parameter concerning contribution of local glucose state to glucose utilization by peripheral tissue in Equation 11	0.001
*k*_5_	Positive parameter concerning contribution of local insulin state to glucose utilization by peripheral tissue in Equation 11	0.001
*k*_6_	Positive parameter concerning contribution of local glucose state to insulin secretion within the pancreatic mass compartment in Equation 18	1.0
*k*_7_	Positive parameter concerning contribution of local glucagon state to insulin secretion within the pancreatic mass compartment in Equation 18	0.01
*k*_8_	Positive parameter concerning contribution of local insulin state to insulin removal from insulin-sensitive tissue in Equation 19	0.03
*k*_9_	Positive parameter concerning contribution of local glucose state to glucagon secretion within the pancreatic mass compartment in Equation 27	0.01
*k*_10_	Positive parameter concerning contribution of local insulin state to glucagon secretion within the pancreatic mass compartment in Equation 27	0.1
	Maximum value of glucose concentration in the brain blood compartment at the steady state in equations 4 and 21	1.0
	Minimum value of glucose concentration in the brain blood compartment at the steady state in equations 4 and 21	0.8
*κ*_G_	Gain in Equation 5	0.3
*κ*_I_	Gain in Equation 22	0.1
*τ*_G_	Time constant in Equation 5	1200
*τ*_I_	Time constant in Equation 22	1200
*a*_cg_	Positive parameter concerning contribution of central glucose state to the controlled error in Equation 30	4.0
*a*_ci_	Positive parameter concerning contribution of central insulin state to the controlled error in Equation 30	0.004
*a*_pg_	Positive parameter concerning contribution of peripheral glucose state to the controlled error in Equation 30	0.02
*k*_*γ*_	Positive parameter concerning signal regulating hepatic glucose production in Equation 31	0.0001
*k*_*β*_	Negative parameter concerning signal regulating insulin secretion in Equation 31	-0.1
*k*_*α*_	Positive parameter concerning signal regulating glucagon secretion in Equation 31	100.0

For hepatic glucose generation and peripheral glucose utilization

For pancreatic insulin secretion and insulin removal

For pancreatic glucagon secretion

For BBB/BCB adaptation

For brain feedback regulation

The aforementioned values are assumed for simulation only. They do not have any physiological meaning and/or are not based on strong evidence.

### Model verification

Simulations are conducted using the model to compute the GIG regulatory responses to bolus, stepwise or continuous intravenous glucose infusion. Simulation profiles are compared with clinical data, quantitatively and/or qualitatively, to verify the model.

### Response to bolus intravenous glucose infusion

**Figure 4:**
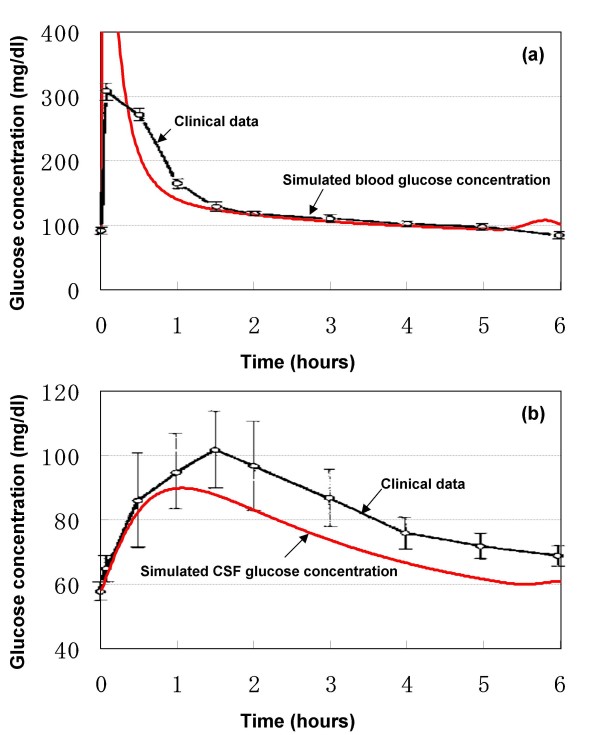
**Response to bolus intravenous glucose infusion**. (a) blood glucose concentration. (b) CSF glucose concentration.

Fishman observed in five normal adult subjects that the CSF glucose level changes in parallel with changes of blood glucose level following intravenous infusion of a bolus of glucose [11]. A similar dose (0.75 mg/kg in 90 seconds) was assumed for the model and the glucose concentration in the arterial blood compartment and that in the CSF compartment were simulated. The results are shown in Fig. 4. The clinical data of Fishman are also shown for comparison [11].

The model-estimated blood glucose concentrations at 0.5, 1, 2, 3 and 4 h were 210.0, 140.0, 116.1, 105.8 and 98.9 mg/dl, respectively (Fig. 4a). The concentration of glucose in the blood increased rapidly during intravenous glucose induction. Following the completion of bolus administration, the concentration decreased rapidly. A plateau concentration was reached at about 4 h.

As shown in Fig. 4b, the model-estimated CSF glucose concentrations increased at first and then decreased during the 6 h simulation. The concentration of glucose in the CSF increased more slowly during induction than it did in the blood and the decrease was also slower than that in the blood. The estimated CSF glucose concentrations at 0.5, 1, 2, 3 and 4 h were 83.0, 89.8, 83.0, 73.6 and 66.5 mg/dl, respectively. Glucose concentration reached a plateau after 5 h. These results demonstrate that the CSF glucose concentration correlates with, and is much lower than blood glucose concentration.

Visual inspection of the data displayed in Fig. 4 shows that both arterial and CSF glucose concentrations estimated by the model following bolus intravenous glucose are comparable with the clinical observations by Fishman [11]. Particularly, a variable time about 4-6 h was required in vivo before the CSF glucose level reached its steady-state equilibrium with the blood glucose. CSF glucose level in the experimental subjects would not reach a peak level for about 2 hours after rapid intravenous glucose injection, and did not reach its equilibrium for about 4 hours. CSF glucose was normally about 65% of blood glucose. The current model simulated the peak CSF glucose concentration at about 1 h and the CSF glucose level continued to decrease for 5 h. A similar CSF-blood glucose ratio of less than 1.0 was also simulated in the current model.

Altogether, the blood and CSF glucose concentrations predicted by the model are compatible to the clinical data concerning the GIG regulatory system response to a bolus intravenous glucose infusion.

### Response to stepwise intravenous glucose injection

**Figure 5:**
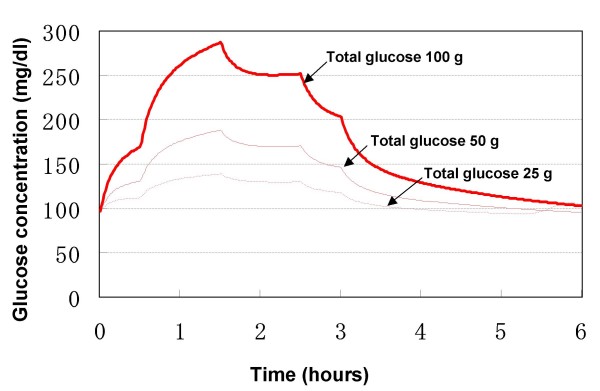
**Blood glucose concentration response to stepwise intravenous glucose infusion**.

Tillil and colleagues measured blood glucose concentrations following intravenous glucose administration in 7 normal subjects, each of whom received 20, 50 and 100 g of glucose intravenously over 3 h [39]. Similar doses were used in the model; thus, 12% of the total glucose dose was assumed to be infused into the venous compartment of the model in the first 30 min, 48 and 32% over the next 2 h, respectively, and 8% over the final 30 min. The simulation results of arterial blood glucose are shown in Fig. 5.

Glucose concentration in the blood increased immediately in response to intravenous glucose infusion. As shown by the first half of the simulation profiles, the resultant blood glucose concentrations, as well as their changes, depended on the infusion rate. The incremental and total areas under the glucose concentration curves increased significantly in response to increasing doses. In each simulation profile, the peak glucose concentration occurred at 1.5 h, i.e., the end of infusion at maximum rate.

These simulation results demonstrated that blood insulin concentration also increased with respect to intravenous glucose infusion. The incremental and total areas under the blood insulin concentration curves increased in parallel with that of blood glucose curves in response to increasing glucose dose (data not shown). The peak insulin concentration occurred after the peak glucose concentration. With increasing doses of intravenous glucose, the blood insulin concentrations were elevated. Even when glucose concentration returned to its basal value at the end of the simulation period, the concentration of insulin in blood was still higher than the basal value.

Tillil and coworkers noted that incremental glucose areas after intravenous glucose injection increased as a function of the glucose dose [39]. The same was true for the incremental and total areas under the insulin concentration curves. The elevated insulin concentration returned to its base value later than the elevated glucose concentration. It was also demonstrated in their normal subjects that with increasing doses of intravenous glucose there was an increase in peripheral blood glucose response as well as in the insulin secretory response.

Considered together, the responses depicted by the model with respect to stepwise intravenous glucose infusion are consistent with the clinical observations.

### Response to continuous intravenous glucose injection

**Figure 6:**
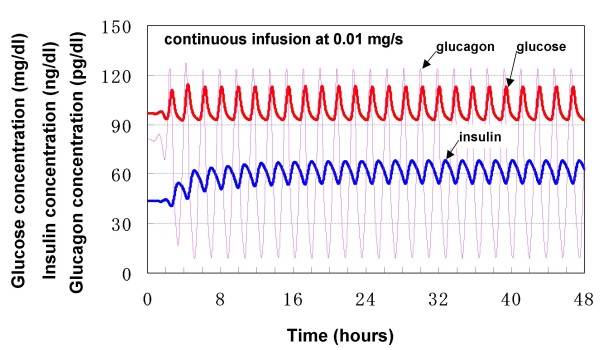
**Response to continuous intravenous glucose infusion**.

Changes in blood concentrations of glucose, insulin and glucagon following continuous intravenous glucose infusion (at 0.01 mg/s) were simulated in the model. As shown in Fig. 6, oscillations were observed in each of the simulation profiles of blood glucose, insulin and glucagon. All periods of oscillation were about 120 min., while their amplitudes were about 20 mg/dl for glucose, 15 ng/dl for insulin and 110 pg/dl for glucagon. It was demonstrated that both glucose and glucagon reached peak levels before that of insulin. The estimated time difference between glucose and insulin peaks was about 30 min.

The model simulation results are consistent with those of currently existing theoretical models, such as the models of Sturis et al. and Li et al. [7,8], both of which were developed to mimic experimental findings of ultradian oscillations during constant glucose infusion. Sturis' model simulated the amplitude of glucose oscillation of 20 mg/dl, as simulated in the current model. Li's model demonstrated that both the oscillation amplitude of blood glucose and that of blood insulin depended on the glucose infusion rate. It was confirmed that the simulated oscillation amplitudes of blood glucose and insulin in the current model were within the amplitude ranges predicted by Li's model.

Both Sturis' and Li's models simulated earlier glucose oscillation than insulin oscillation. For example, Li's model predicted the time difference between glucose and insulin peak levels to be about 20 min, which also depended on the glucose infusion rate. The simulation results in our model are in agreement with these reported results.

With respect to the effect of glucose infusion rate on ultradian oscillation, it was demonstrated in Li's model that there existed a bifurcation point for the glucose infusion rate (about 1.25 mg/dl/min or 2 mg/s if the volume of glucose space is 10 liters [7]. Therefore, the oscillations in blood glucose concentrations were sustained when the rate of infused glucose was somewhat less than the bifurcation point. Particularly, ultradian oscillation would be sustained theoretically by a little glucose infusion. When the rate of infused glucose was larger than the bifurcation point, no oscillation would be sustained. It should be pointed out that the oscillations arise even without glucose infusion in the Li's model, majorly because the oscillation is induced by two explicit time delays.

**Figure 7:**
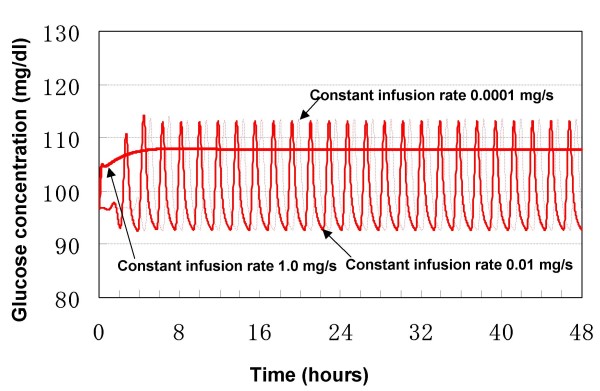
**Dependence of ultradian oscillation on glucose infusion rate**.

The current developed model was used to simulate how changes in glucose infusion rate affect the ultradian oscillation. Fig. 7 shows that oscillation would occur even when the rate of intravenous glucose was 0.0001 mg/s while it would disappear when the constant glucose infusion rate was 1.0 mg/s. Furthermore, the bifurcation point for the glucose infusion rate in this model is estimated to be about 0.4374 mg/s. Although this identified bifurcation point is somewhat different from that of Li's model, the current model is comparable with Li's model in simulating the existence of Hopf bifurcation point for glucose infusion rate.

Taken together, our model is consistent with the currently existing models in describing ultradian oscillations, based on their quantitative and qualitative agreements.

### Other qualitative verifications

#### Response to bolus intravenous insulin infusion

Simulated infusion of insulin (375 ng/kg over 90 seconds) into the venous compartment resulted in increases in both blood and CSF insulin levels, while blood glucose level was maintained between 90 and 120 mg/dl. The simulation results demonstrated that insulin concentration dynamics in the blood and that in the CSF are characteristic of two- or three-compartmental model, and are consistent with the results reported by Schwartz and coworkers [40], where dynamics of CSF insulin was described by a three-compartment model.

#### Response to continuous intracranial glucose infusion

To examine the central effects of glucose on systemic glucose homeostasis, Lam and colleagues infused glucose directly into the third cerebral ventricle of conscious rats [2]. The results showed that such infusion lowered blood glucose levels. In our model, simulated glucose infusion into CSF compartment decreased blood glucose concentration. However, the estimated level of blood glucose was dependent on the infusion rate; a small rate (0.05 mg/s) resulted in ultradian oscillations in blood glucose, while a large infusion rate (0.25 mg/s) resulted in the disappearance of such oscillations.

#### Response to continuous intracranial insulin infusion

Woods and colleagues suggested that insulin signaling in the hypothalamus initiates a signal via the vagus nerve to the liver to reduce glucose synthesis and secretion into the blood [30]. Hence, insulin in the brain acts by reducing peripheral blood glucose levels. In our model, simulated insulin infusion at rates between 20 and 30 ng/s into CSF compartment decreased blood glucose levels. The resultant blood glucose concentration was dependent on the infusion rate. That is, the higher the infusion rate, the lower the resultant blood glucose level.

Altogether, the results of simulation studies demonstrate that intracranial infusion of glucose or insulin results in reduction of peripheral blood glucose levels.

The above simulation results (which are summarized in Table 5) provide solid support and verification of our model.

**Table 5:** Compatibility of results of model simulation with clinical data.

Item	Input of model	Reference
CSF glucose dynamics	Bolus intravenous glucose infusion	[11]
Blood glucose dynamics and blood insulin dynamics	Stepwise intravenous glucose infusion	[39]
Ultradian oscillation	Continuous intravenous glucose infusion	[7,8]
Bifurcation point of ultradian oscillation	Continuous intravenous glucose infusion	[8]
CSF insulin dynamics	Bolus intravenous insulin infusion	[40]
Blood glucose dynamics	Continuous intracranial glucose infusion	[2]
Blood glucose dynamics	Continuous intracranial insulin infusion	[30]

## Results

To determine the roles of stress and BBB adaptation in the generation of hyperglycemia, which generally accompanies hyperinsulinemia and hyperglucagonemia in diabetes, simulated stress input of varying strength and duration was introduced in the model, together with fast BBB adaptation. The use of various simulation paradigms allowed clarification of the relationship between brain glucose homeostasis and blood hyperglycemia in diabetes.

### Role of stress

As described during modeling, stress, which is described abstractly by parameter *s *in eq. (10"), (18") and (27"), is a disturbance input to the central controller. It affects the efferent signals that regulate hepatic glucose production and pancreatic hormonal secretion in this model. The parameter *s *varies from 0 to 1, which describes the severity of stress from mild to severe. Furthermore, the duration of stress (short-term or long-term) can also affect glucose production. Time constant of BBB adaptation in this simulation is of 1200 h.

**Figure 8:**
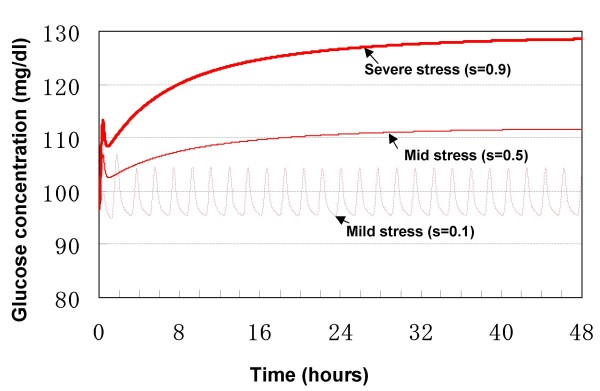
**Response to long-term stress of variable severity**.

**Figure 9:**
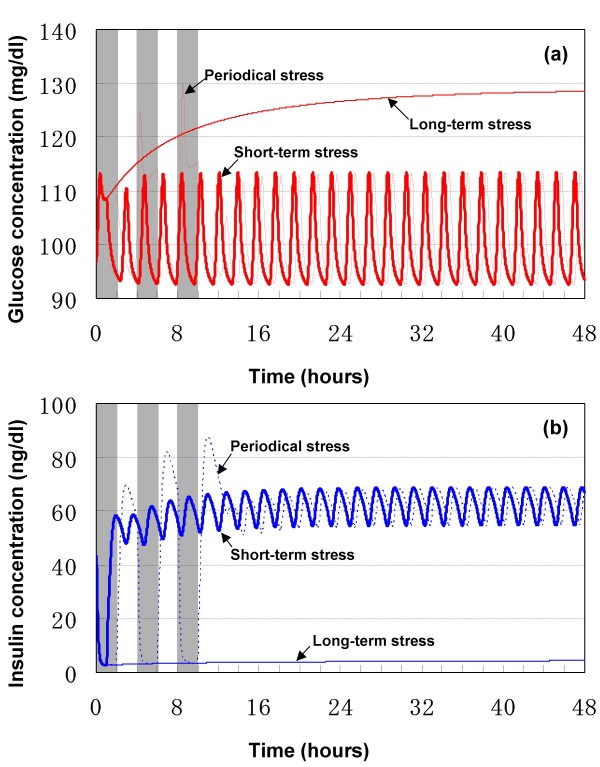
**Response to severe stress of variable duration**. (a) blood glucose concentration. (b) blood insulin concentration.

The results of simulated stress are shown in Figs. 8 and 9. Stress, independent of its duration and strength, caused transient or persistent peripheral hyperglycemia in all simulations.

With regard to the severity/duration of stress, a long-term mild stress, corresponding to s = 0.1, resulted in a steady-state peripheral hyperglycemia characteristic of ultradian oscillation, in the GIG regulatory system (Fig. 8). In contrast, the ultradian oscillation disappeared upon simulation of mid- (s = 0.5) to severe-stress (s = 0.9). The Hopf bifurcation point for stress is estimated to be about 0.24. That is to say, if stress is smaller than 0.24 (s < 0.24), the oscillations are sustained and if stress is larger than 0.24 (s > 0.24), no oscillation will be sustained.

The level of resultant hyperglycemia was dependent on the severity of stress, i.e., the worst the stress, the higher the resultant blood glucose level. Continuous application of severe stress (s = 0.9) resulted in persistent hyperglycemia and blood glucose never returned to its basal level, rather, a new steady-state hyperglycemia (> 120 mg/dl) was established. On the other hand, blood glucose level was still within the euglycemic range (< 120 mg/dl) following imposition of mid-level stress (s = 0.5). Considered together, mild-to-mid level stress seems to induce little disturbance of the GIG regulatory system, while severe stress has the worst influence.

With regard to the duration of stress, short-term severe stress (s = 0.9) applied in the first 1 h caused a transient increase in blood glucose level (Fig. 9a), and decreases in blood insulin (Fig. 9b) and blood glucagon levels (data not shown). These transient responses disappeared after removal of stress during the simulated 48 hours. That is to say, there is no bifurcation point for the length of time over which the short-term stress is applied. Short-term stress resulted in a steady-state, characteristic of ultradian oscillation, together with elevated blood insulin and glucagon.

Recursive stress of 4-hour period (2 h active, as shown by the shaded areas in Fig. 8, and then 2 h inactive) was applied. The simulation results showed a continuous increase in maximum blood glucose during the application of such stress. The insulin concentration was depressed during each active period but rebounded during each inactive period. The peak insulin concentrations were elevated during stress application, with a time-delay compared to peak glucose concentration. Such increase and time delay are probably due to the inertia of the GIG regulatory system. Similar to that of short-term stress, responses of blood glucose to repeated stress also converged to a similar steady-state following termination of stress.

Although human studies on the role of stress on the onset and course of diabetes are limited, a large body of evidence based on animal studies supports the notion that stress reliably induces hyperglycemia in diabetes [4]. Importantly, the results of simulation tests using the present model compare well with clinical knowledge. Therefore, the model is partially verified in modeling the effects of stress on the generation of hyperglycemia. Quantification of severity of stress using parameter *s *varying from 0 to 1 seems a reasonable approach.

### Role of BBB adaptation

**Figure 10:**
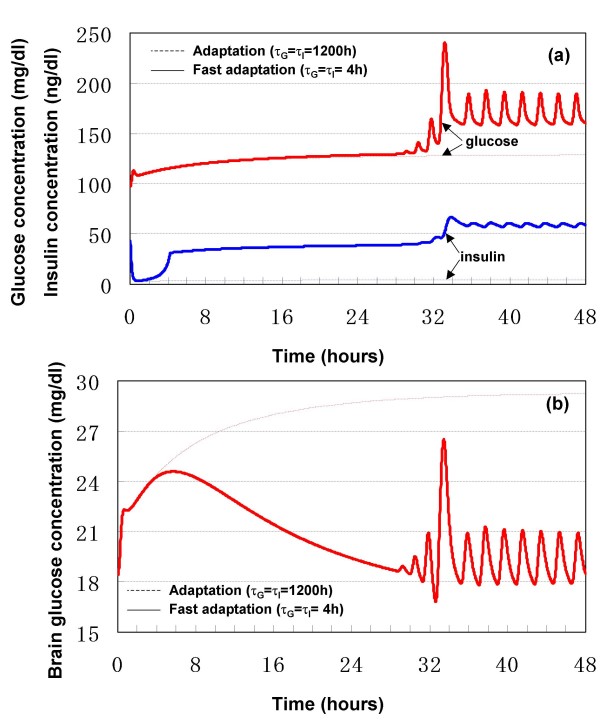
**Response to long-term severe stress with BBB adaptation**. (a) blood concentrations of glucose and insulin. (b) brain glucose concentration.

In order to simulate the roles of BBB adaptation in the generation of hyperglycemia, the time constants in equations (5) and (22) were assumed to be 4 h in the model, although these time constants may be some years for an adapting BBB in human. Such an assumption of short time constant is due to the time limitation of computer simulation. Therefore, the simulation results (solid line in Fig. 10) should be considered as fast-forward of the responses (broken line). Long-term severe stress (s = 0.9) is assumed in the simulation.

As shown in Fig. 10a, with a fast BBB adaptation, a hyperglycemic steady-state of blood glucose was generated in the present model, while brain glucose was maintained within the euglycemic range. At the same time, both the concentrations of blood insulin and blood glucagon were higher than their basal values, while the ratio of glucagon to insulin was elevated. The present model simulated an abnormal state, which is similar to diabetes/hyperglycemia, hyperinsulinemia and hyperglucagonemia [32,41], of the GIG regulatory system under long-term stress, together with a fast BBB adaptation.

The simulation results are consistent with clinical facts. That is, as reviewed by Jiang and Zhang [42], the absolute levels of glucagon or the ratios of glucagon to insulin are often elevated in various forms of diabetes in both animal and human subjects. The same authors also pointed out that chronic hyperglucagonemia correlated with and was in part responsible for increased hepatic glucose production and hyperglycemia in type 2 diabetes. Based on the physiological fact that stress increases glucagon secretion through the HPA axis, it is reasonable to consider stress as one of the causes of hyperglycemia in diabetes. As shown in Fig. 10b, brain glucose homeostasis was achieved during peripheral hyperglycemia. The brain glucose is at euglycemic level while blood glucose is at hyperglycemic level. The simulation result should motivate clinical verification whether chronic hyperglycemia in diabetic patients does not alter brain glucose concentrations, compared with the healthy subjects.

In order to simulate the effect of gain of BBB adaptation on the resultant hyperglycemia, *κ*_*G *_in equation (5) was varied from 0 to 0.5. The simulation results showed that the resultant peripheral hyperglycemia was dependent on *κ*_*G*_. That is, the larger the gain *κ*_*G*_, the worse the resultant hyperglycemia (data not shown). If *κ*_*G *_= 0, which corresponds to an inadaptive BBB, a state of elevated glucose levels in brain and blood within the euglycemic range would be inevitable. However, the elevated brain glucose would never return to its basal level.

These simulation results demonstrated that BBB adaptation contributes to the generation of hyperglycemia and homeostasis of brain glucose level under stress. Both the gain and time constant of BBB adaptation to dysglycemia are important. Based on their dependence on the individual, it is reasonable to speculate that a person with a large gain and short time constant would be sensitive to stress-induced hyperglycemia and ultimately develops diabetes.

## Discussion

### Importance of brain glucose homeostasis

The brain is one of the major organs that require continuous energy supply by glucose. Maintenance of constant glucose concentration in the brain is of supreme importance mainly due to the fact that the brain is uniquely dependent on the availability of glucose and that dysglycemia, either hypoglycemia or hyperglycemia, would induce brain dysfunction. Not only in healthy individuals but also in diabetics, brain glucose concentration is maintained at constant level. Physiological evidence suggests that the maintenance of constant glucose level in the brain is more important than that in the blood.

The present study was based on the theme that the ultimate goal of glucose-insulin-glucagon (GIG) regulatory system is not blood glucose homeostasis, but rather brain glucose homeostasis. Anatomically, different parts of the brain, particularly the hypothalamus, are important centers involved in the regulation of brain glucose homeostasis. Physiologically, changes in brain glucose levels elicit a complex neuroendocrine response that rapidly corrects dysglycemia in the brain.

Whereas the relations among the brain, glucose homeostasis and diabetes have been qualitatively recognized in experimental animals and clinically in patients, no theoretical analysis is available on the relationship between brain glucose homeostasis and hyperglycemia in diabetes. To the best of our knowledge, this is the first paper that targets the control of brain glucose homeostasis and provides a theoretical framework for the role of the brain in the generation of hyperglycemia in diabetes.

### Role of stress

Stress has long been shown to have major effects on metabolic activity as energy mobilization is a primary result of the fight-or-flight response. Stress stimulates the release of various hormones, which can increase blood glucose levels. Based on the same mechanism, stress could also potentially induce a state of chronic hyperglycemia in diabetes. Although human studies on the role of stress on the onset and course of diabetes are limited, the notion that stress reliably produces hyperglycemia in disease has been supported by animal studies [4].

Since no quantitative measure of stress is available at present, an abstract variable of positive value varying between 0 and 1 was introduced in the model to describe the severity of stress from mild to severe. The stress-simulation results compared well with clinical knowledge concerning the stress-induced hyperglycemia in diabetes [4]. Various simulations of the severity and duration of stress conducted in the present study emphasized the roles of stress in the appearance of hyperglycemia, not only qualitatively but also quantitatively. As a corollary to these simulation results, some patients with diabetes may not require glucose-lowering agents at all, rather, they may benefit from a less obvious treatment directed against stress, such as relaxation.

### BBB adaptation as a main cause of diabetes

To maintain brain glucose homeostasis, glucose transport from the blood to the brain should be regulated accurately, according to the short-term and long-term states of blood glucose. In fact, BBB helps govern the brain glucose homeostasis through temporary and permanent mechanisms. The former is through the BBB semi-permeability to glucose, which is a physical filter with respect to sharp swings in blood glucose levels, while the latter involves BBB adaptation, which is a physiological change with respect to chronic hypoglycemia or hyperglycemia.

As shown by the simulation results, BBB adaptation for brain glucose homeostasis, together with long-term severe stress, contributes to hyperglycemia. A vicious cycle of hyperglycemia and BBB adaptation would exist in diabetes. However, such a feedback loop should be considered as a self-protective mechanism of the brain, through which brain glucose homeostasis is maintained. In other words, brain glucose homeostasis requires increasing blood glucose levels if glucose transport from the blood to the brain is depressed due to BBB adaptation. If not, the brain would be short of glucose, as seen in "insulin shock", where the blood glucose is controlled euglycemically by insulin while the glucose transport across the adapted BBB is not improved.

Theoretical analysis suggests a novel hypothesis, namely, hyperglycemia observed in diabetes is one of the controlled results for brain glucose homeostasis through a permanent adaptation mechanism. Also, as a suggestion, re-modification of BBB adaptation in diabetes, with pharmacokinetic or genetic measures, could be a potentially suitable strategy for clinical control of hyperglycemia in diabetes.

### Modeling of ultradian oscillation

Although our understanding of diabetes has progressed very rapidly in the past decades, diabetes is still a disease that has no cure. Every effort, both in vivo and in vitro, should be made to prevent and treat this disease. In this regard, theoretical or mathematical models constitute interesting tools, because they can provide insights, improve intuitions, clarify assumptions of formal theory, allow for estimating parameters, determining sensitivities, simulating simple and complex phenomena and providing future predictions for diabetes control.

The current model was particularly successful in providing a novel interpretation of the mechanism underlying ultradian oscillation. The hypothesis that ultradian insulin secretion represents an instability in peripheral GIG interactions has been the subject of a number of studies, including the mathematical model originally proposed by Sturis et al. and lately modified by Li et al. [7,8], where time-delay is included implicitly or explicitly to produce ultradian oscillation.

The present model has similar characteristics of the models of Sturis et al. and Li et al. [7,8], in simulating ultradian oscillation, without including any time-delay. It is considered that oscillation is generated in the model mainly due to feedback control via peripheral GIG interactions and the central brain-endocrine crosstalk, but not due to time-delay. The model does not exclude the possible role of time-delay in generating ultradian oscillation, as permeability at the blood-mass interface may introduce some implicit time-delay and partially contribute to the generation of ultradian oscillation in the model.

### Limitations of the model

No model ever reproduces all facets of the original system. As pointed by Gatewood and colleagues [43], no matter how small pieces one chooses, there always seems to be smaller ones; no matter how many details one models, there are always others. The same applies to modeling the GIG regulatory system. In this paper, the brain glucose homeostasis is targeted and modeled as the principle of GIG regulatory system. The relationship between brain, as well as stress, and hyperglycemia in diabetes is simulated in the model. Although the simulation results are highly suggestive, modeling is based on some assumptions.

One of the important limitations of the model is the need of a set of physical and physiological parameters that is difficult or impossible to estimate within a single individual. Although various values are given for most of these parameters based on the literature, more than 20 parameters have to be given arbitrary values selected by the authors. Verifications given in the section of model verification provide greater assurance that predictions made by the model are correct. However, the model only simulates an "average subject". A formal sensitivity analysis is left for future research.

Further testing of the model is also required to determine whether it is suitable for individual patient parameterization which is a key requirement for clinical use. Further refinements might be appropriate after tuning various parameters to fit pertinent clinical data. However, the model in its current form clearly has a role as a patient simulator. In this respect, it provides theoretical framework with which to analyze the relationship between brain glucose homeostasis and blood hyperglycemia in diabetes and to plan therapeutic strategies for diabetes.

## Conclusion

In this paper, the control of brain glucose homeostasis was considered as the ultimate goal of the glucose-insulin-glucagon (GIG) regulatory system. In order to demonstrate theoretically the relationship between brain glucose homeostasis and hyperglycemia in diabetes, a brain-centered compartment model of GIG regulatory system is developed from the viewpoint of systems control. The model consists of both peripheral GIG interactions and central brain-endocrine crosstalk, while taking account of the effects of stress and BBB adaptation to dysglycemia.

The results of various simulations highlighted the unique features of the GIG regulatory system. In particular, an abnormal state similar to diabetes, characterized by blood hyperglycemia but brain glucose homeostasis, was simulated in the model of a person with a fast BBB adaptation and under long-term severe stress. Based on the simulation results, we conclude that, (i) both long-term severe stress and BBB adaptation contribute to hyperglycemia; (ii) blood hyperglycemia may be an outcome of control of brain glucose homeostasis in diabetes. Based on these theoretical results, *in vivo *experiments are welcome to provide direct evidence for the relationship between brain glucose homeostasis and hyperglycemia in diabetes.

## Abbreviations

*E*: glucagon concentration; **E**: vector of glucagon concentration; *G*: glucose concentration; **G**: vector of glucose concentration; *I*: insulin concentration; **I**: vector of insulin concentration; *K*: Michaelis constant of Michaelis-Menten equation; *M*: metabolism; *T*: maximal transport rate of Michaelis-Menten equation; *V*: distribution volume; **V**: matrix of distribution volume; W: vector of blood flow; *a*: estimable parameter in equation (30); *f*: transfer rate among the adjacent compartments; *h*: permeability coefficient; *k*: estimable parameters in equations (10), (11), (18), (19), (27) and (31); *m*: estimable parameters in equation (2); *p*: production rate; *s*: strength of stress; *t*: time; *u*: utilization rate; *v*: degradation constant of glucagon; *w*: blood flow or urine flow; *α*: regulatory signal of glucagon secretion; *β*: regulatory signal of insulin secretion; *γ*: regulatory signal of hepatic glucose production; *κ*: gain; *τ*: time constant.

### Subscript

0: value at the steady-state; *E*: glucagon; *G*: glucose; *I*: insulin; *art*: arterial blood or arterial part of cardiocirculatory segment; *blood*: blood compartment; *cg*: central information of glucose; *ci*: central information of insulin; *csf*: cerebrospinal fluid (CSF) compartment; *error*: controlled error; *mass*: mass compartment; *pg*: peripheral information of glucose; *ven*: venous blood or venous part of cardiocirculatory segment; *E-blood-mass*: glucagon transport from the blood compartment to the mass compartment; *G-blood-csf*: glucose transport from the blood compartment to the CSF compartment; *G-csf-mass*: glucose transport from the CSF compartment to the brain mass compartment; *G-blood-mass*: glucose transport from the blood compartment to the mass compartment; *I-blood-mass*: insulin transport from the blood compartment to the mass compartment.

### Superscript

*brain*: brain (cranial) segment; *inf*: exogenous infusion; *lung*: lung segment; *liv*: liver segment; *pan*: pancreas segment; *red*: red blood cells; *urine*: urine.

## Competing interests

The authors declare that they have no competing interests.

## Authors' contributions

The authors contributed equally to this work. All authors read and approved the final manuscript.

## References

[B1] Sandoval D Cota D Seeley RJ The integrative role of CNS fuel-sensing mechanisms in energy balance and glucose regulation Annu Rev Physiol 2008 70 513 535 10.1146/annurev.physiol.70.120806.095256 17988209

[B2] Lam TK Gutierrez-Juarez R Pocai A Rossetti L Regulation of blood glucose by hypothalamic pyruvate metabolism Science 2005 309 943 947 10.1126/science.1112085 16081739

[B3] Gjedde A Crone C Blood-brain glucose transfer: repression in chronic hyperglycemia Science 1981 214 456 457 10.1126/science.7027439 7027439

[B4] Surwit RS Schneider MS Feinglos MN Stress and diabetes mellitus Diabetes Care 1992 15 1413 1422 10.2337/diacare.15.10.1413 1425110

[B5] Bolie VW Coefficients of normal blood glucose regulation J Appl Physiol 1961 16 783 788 13870789 10.1152/jappl.1961.16.5.783

[B6] Bergman RN Ider YZ Bowden CR Cobelli C Quantitative estimation of insulin sensitivity Am J Physiol 1979 236 E667 677 443421 10.1152/ajpendo.1979.236.6.E667

[B7] Sturis J Polonsky KS Mosekilde E Van Cauter E Computer model for mechanisms underlying ultradian oscillations of insulin and glucose Am J Physiol 1991 260 E801 809 2035636 10.1152/ajpendo.1991.260.5.E801

[B8] Li J Kuang Y Mason CC Modeling the glucose-insulin regulatory system and ultradian insulin secretory oscillations with two explicit time delays J Theor Biol 2006 242 722 735 10.1016/j.jtbi.2006.04.002 16712872

[B9] Boutayeb A Chetouani A A critical review of mathematical models and data used in diabetology Biomed Eng Online 2006 5 43 16808835 10.1186/1475-925X-5-43 PMC1553453

[B10] Makroglou A Li J Kuang Y Mathematical models and software tools for the glucose-insulin regulatory system and diabetes: an overview Appl Numer Math 2006 56 559 573 10.1016/j.apnum.2005.04.023

[B11] Fishman RA Cerebrospinal fluid in diseases of the nervous system 1992 W.B. Saunders Company, Pennsylvania

[B12] Aszalós Z [Cerebral complications of diabetes mellitus] [Article in Hungarian] Orv Hetil 2007 148 2371 2376 10.1556/OH.2007.28221 18055361

[B13] Routh VH Glucose-sensing neurons: are they physiologically relevant? Physiol Behav 2002 76 403 413 10.1016/S0031-9384(02)00761-8 12117577

[B14] Silver IA Erecinska M Glucose-induced intracellular ion changes in sugar-sensitive hypothalamic neurons J Neurophysiol 1998 79 1733 1745 9535943 10.1152/jn.1998.79.4.1733

[B15] Levin BE Kang L Sanders NM Dunn-Meynell AA Role of Neuronal Glucosensing in the Regulation of Energy Homeostasis Diabetes 2006 55 S122 130 10.2337/db06-S016

[B16] Banks WA The source of cerebral insulin Eur J Pharmacol 2004 490 5 12 10.1016/j.ejphar.2004.02.040 15094069

[B17] Uyama N Geerts A Reynaert H Neural connections between the hypothalamus and the liver Anat Rec A Discov Mol Cell Evol Biol 2004 280 808 820 10.1002/ar.a.20086 15382020

[B18] Gaohua L Kimura H A mathematical model of intracranial pressure dynamics for brain hypothermia treatment J Theor Biol 2006 238 882 900 10.1016/j.jtbi.2005.06.036 16098539

[B19] Gaohua L Kimura H Simulation of propofol anaesthesia for intracranial decompression using brain hypothermia treatment Theor Biol Med Model 2007 4 46 10.1186/1742-4682-4-46 18045501 PMC2217543

[B20] Gaohua L Kimura H A mathematical model of respiratory and biothermal dynamics in brain hypothermia treatment IEEE Trans Biomed Eng 2008 55 1266 1278 10.1109/TBME.2007.912400 18390318

[B21] Gaohua L Maekawa T Kimura H An integrated model of thermodynamic-hemodynamic-pharmacokinetic system and its application on decoupling control of intracranial temperature and pressure in brain hypothermia treatment J Theor Biol 2006 242 16 31 10.1016/j.jtbi.2006.01.033 16524597

[B22] Guyton AC Hall JE Textbook of Medical Physiology 2001 W.B. Saunders Company, Pennsylvania

[B23] Rapoport SI Blood-Brain Barrier in Physiology and Medicine 1976 Raven Press, New York

[B24] Simpson IA Appel NM Hokari M Oki J Holman GD Maher F Koehler-Stec EM Vannucci SJ Smith QR Blood-brain barrier glucose transporter: effects of hypo- and hyperglycemia revisited J Neurochem 1999 72 238 247 10.1046/j.1471-4159.1999.0720238.x 9886075

[B25] Criego AB Tkac I Kumar A Thomas W Gruetter R Seaquist ER Brain glucose concentrations in healthy humans subjected to recurrent hypoglycemia J Neurosci Res 2005 82 525 530 10.1002/jnr.20654 16235252

[B26] Cerasi E An analogue computer model for the insulin response to glucose infusion Simulation 1971 16 243 255 10.1177/003754977101600601 6071595

[B27] Bodenlenz M Schaupp LA Druml T Sommer R Wutte A Schaller HC Sinner F Wach P Pieber TR Measurement of interstitial insulin in human adipose and muscle tissue under moderate hyperinsulinemia by means of direct interstitial access Am J Physiol 2005 289 E296 300 10.1152/ajpendo.00431.2004 15769794

[B28] Gudbjörnsdóttir S Sjöstrand M Strindberg L Wahren J Lönnroth P Direct measurements of the permeability surface area for insulin and glucose in human skeletal muscle J Clin Endocrinol Metab 2003 88 4559 4564 10.1210/jc.2003-030434 14557422

[B29] Baura GD Foster DM Porte D Jr Kahn SE Bergman RN Cobelli C Schwartz MW Saturable transport of insulin from plasma into the central nervous system of dogs in vivo. A mechanism for regulated insulin delivery to the brain J Clin Invest 1993 92 1824 1830 10.1172/JCI116773 8408635 PMC288346

[B30] Woods SC Benoit SC Clegg DJ The Brain-Gut-Islet Connection Diabetes 2006 55 S114 121 10.2337/db06-S015

[B31] Puglianiello A Cianfarani S Central control of glucose homeostasis Rev Diabet Stud 2006 3 54 60 10.1900/RDS.2006.3.54 17487327 PMC1783580

[B32] Kahn CR Weir GC King GL Jacobson AM Moses AC Smith RJ Joslin's Diabetes Mellitus 2005 Lippincott Williams & Wilkins, Philadelphia

[B33] Woods SC Porte D Jr Bobbioni E Ionescu E Sauter JF Rohner-Jeanrenaud F Jeanrenaud B Insulin: its relationship to the central nervous system and to the control of food intake and body weight Am J Clin Nutr 1985 42 1063 1071 3904396 10.1093/ajcn/42.5.1063

[B34] Sonksen P Sonksen J Insulin: understanding its action in health and disease Br J Anaesth 2000 85 69 79 10.1093/bja/85.1.69 10927996

[B35] Fisher M Sherwin RS Hendler R Felig P Kinetics of glucagon in man: effects of starvation Proc Natl Acad Sci USA 1976 73 1735 1739 10.1073/pnas.73.5.1735 1064045 PMC430375

[B36] Regittnig W Ellmerer M Fauler G Sendlhofer G Trajanoski Z Leis HJ Schaupp L Wach P Pieber TR Assessment of transcapillary glucose exchange in human skeletal muscle and adipose tissue Am J Physiol Endocrinol Metab 2003 285 E241 251 12684220 10.1152/ajpendo.00351.2002

[B37] Eng J Yalow RS Evidence against extrapancreatic insulin synthesis Proc Natl Acad Sci USA 1981 78 4576 4578 10.1073/pnas.78.7.4576 6270683 PMC319835

[B38] Curry FR Atrial natriuretic peptide: an essential physiological regulator of transvascular fluid, protein transport, and plasma volume J Clin Invest 2005 115 1458 1461 10.1172/JCI25417 15931381 PMC1137012

[B39] Tillil H Shapiro ET Miller MA Karrison T Frank BH Galloway JA Rubenstein AH Polonsky KS Dose-dependent effects of oral and intravenous glucose on insulin secretion and clearance in normal humans Am J Physiol 1988 254 E349 357 3279811 10.1152/ajpendo.1988.254.3.E349

[B40] Schwartz MW Bergman RN Kahn SE Taborsky GJ Fisher LD Sipols AJ Woods SC Steil GM Porte D Evidence for entry of plasma insulin into cerebrospinal fluid through an intermediate compartment in dogs. Quantitative aspects and implications for transport J Clin Invest 1991 88 1272 1281 10.1172/JCI115431 1918377 PMC295596

[B41] Reaven GM Chen YD Golay A Swislocki AL Jaspan JB Documentation of hyperglucagonemia throughout the day in nonobese and obese patients with noninsulin-dependent diabetes mellitus J Clin Endocrinol Metab 1987 64 106 110 10.1210/jcem-64-1-106 3536980

[B42] Jiang G Zhang BB Glucagon and regulation of glucose metabolism Am J Physiol 2003 284 E671 678 10.1152/ajpendo.00492.2002 12626323

[B43] Gatewood LC Ackerman E Rosevear JW Molnar GD Modeling blood glucose dynamics Behav Sci 1970 15 72 87 10.1002/bs.3830150108 5413469

